# Garlic Peel-Derived Phytochemicals Using GC-MS: Antioxidant, Anti-Inflammatory, and Anti-Apoptotic Effects in Ulcerative Colitis Rat Model

**DOI:** 10.3390/ph18070969

**Published:** 2025-06-27

**Authors:** Duaa A. Althumairy, Rasha Abu-Khudir, Afnan I. Alandanoosi, Gehan M. Badr

**Affiliations:** 1 Department of Biological Sciences, College of Science, King Faisal University, P.O. Box 380, Al-Ahsa 31982, Saudi Arabia; dalthumairy@kfu.edu.sa; 2 Department of Chemistry, College of Science, King Faisal University, P.O. Box 380, Al-Ahsa 31982, Saudi Arabia; 3 Department of Food and Nutrition Sciences, College of Agricultural and Food Sciences, King Faisal University, P.O. Box 380, Al-Ahsa 31982, Saudi Arabia; aalandanoosi@kfu.edu.sa; 4 Department of Zoology, Faculty of Science, Ain Shams University, Cairo 11566, Egypt; gbadr@sci.asu.edu.eg

**Keywords:** ulcerative colitis, garlic peel extract, antioxidants, anti-inflammatory, cytokines, GC-MS, fatty acid esters, natural therapy

## Abstract

**Background/Objectives**: Ulcerative colitis (UC) is a chronic, relapsing inflammatory bowel disease (IBD) that poses a significant gastroenterological challenge. **Methods**: This study investigates the protective effects of garlic peel extract (GPE) in a rat model of acetic acid (AA)-induced colitis. Rats received oral GPE (100 mg/kg) for 14 days prior to AA administration, and this continued for 14 days post-induction. **Results**: GC-MS analysis of GPE identified several key phytochemicals, primarily methyl esters of fatty acids (62.47%), fatty acids (10.36%), fatty acid derivatives (6.75%), and vitamins (4.86%) as the major constituents. Other notable compounds included steroids, natural alcohols, organosulfur compounds, fatty aldehydes, carotenoids, sugars, and glucosinolates. GPE treatment significantly improved body weight and colon length. Biochemical analysis showed that GPE downregulated the levels of the pro-inflammatory cytokines interleukin-1 (IL-1), IL-6, IL-17, tumor necrosis factor-alpha (TNF-α), and nuclear factor-kappa B (NF-κB), compared to the colitis (AA) group. Additionally, GPE reduced the oxidative stress (OS) biomarkers, including myeloperoxidase (MPO) and malondialdehyde (MDA), as well as caspase-3, a marker for apoptosis. Furthermore, GPE treatment resulted in enhanced activities of the enzymatic antioxidants catalase (CAT) and superoxide dismutase (SOD), along with increased levels of the anti-inflammatory cytokine IL-10. These findings were supported by histological evidence. **Conclusions**: Collectively, GPE holds promise as a therapeutic strategy for UC, owing to its natural bioactive compounds and their potential synergistic anti-inflammatory, antioxidant, and anti-apoptotic effects.

## 1. Introduction

Ulcerative colitis (UC) is a chronic, nonspecific inflammatory bowel disease (IBD) that primarily affects the rectum and colon. It typically involves only the mucosal layer, manifesting as continuous ulcerations accompanied by an inflammatory cascade driven by dysregulated innate immune responses and an imbalance between pro- and anti-inflammatory activities [1,2]. The severity of ulceration correlates with immune activation and elevated levels of pro-inflammatory cytokines. Chronic inflammation disrupts the mucosal barrier and activates the nuclear factor-kappa B (NF-κB) signaling pathway, a central regulator of inflammatory responses. NF-κB promotes the expression of cytokines such as IL-1, IL-6, IL-17, and TNF-α, which contribute to tissue damage and mucosal injury [3,4,5,6]. In addition, infiltration of neutrophils and macrophages enhances the production of pro-inflammatory mediators, whereas it downregulates the expression of anti-inflammatory cytokines, including IL-10 [7,8,9]. Moreover, it promotes myeloperoxidase (MPO) release by increasing reactive oxygen species (ROS) levels, which are central to UC development [10,11]. In addition, the observed activation of macrophages and neutrophils in UC leads to the generation of nitric oxide (NO) that triggers lipid peroxidation (LPO) and excessive production of malondialdehyde (MDA) [7]. Oxidative stress (OS) is known to be an important trigger of apoptosis that plays a crucial role in the initiation as well as the progression of UC. This is accompanied by upregulation of apoptotic proteinssuch as caspase-3, caspase-9, and Bax, as well as downregulation of Bcl-2 [12,13,14].

The exact etiology of UC remains unclear, and current therapeutic options are limited. Conventional treatments that primarily target symptom reduction are associated with adverse side effects as well as challenging long-term safety and efficacy. These include 5-aminosalicylates (5-ASAs), corticosteroids (CSs), immunomodulators (IMMs), and biologics, which encompass inhibitors of pro-inflammatory cytokines and antagonists of integrin [15,16,17,18]. Hence, identifying alternative therapeutic modalities that are both efficient and have fewer side effects is of growing interest.

Naturally derived compounds with antioxidant and anti-inflammatory activities have gained attention as plausible alternatives to conventional therapy. Garlic (*Allium sativum*) possesses diverse bioactivities, including antioxidant, anti-inflammatory, antidiabetic, antihypertensive, antimicrobial, and anticancer properties. These biological activities are primarily attributed to organosulfur-containing compounds (OSCs) such as S-allyl-cysteine sulfoxide (alliin), diallyl thiosulfonate (allicin), S-allyl-L-cysteine (SAC), and diallyl sulfide (DAS) [19,20,21,22]. While much attention has been focused on the medicinal properties of garlic bulbs, less is known about the potential benefits of garlic peel, which is often discarded as waste.

Recent studies suggest that garlic peel contains bioactive compounds that may offer significant health benefits, including the modulation of immune responses and the reduction in OS, both of which are crucial in the management of UC [23,24,25,26]. Garlic peel extract (GPE) contains abundant phenolic compounds and exhibits strong antioxidant and antimicrobial activities, comparable or superior to fresh garlic extracts [25,27]. Studies have identified multiple bioactive phenols (phenylpropanoids) in garlic peels that contribute to their antioxidant potential and anticancer effects [23,27,28,29]. Given that UC is associated with altered gut microbiota, immune dysregulation, and compromised mucosal integrity [30,31], GPE’s antimicrobial and immunomodulatory effects may offer therapeutic benefits. Garlic peels also show antifungal properties and potential in modulating fungal microbiota, which are increasingly recognized in the pathology of UC [25,32]. Furthermore, garlic peels are rich in polyphenols, including flavonoids and phenolic acids that suppress COX-2 expression, reduce ROS levels, and exert anti-apoptotic effects by downregulating NF-κB and caspase-3 transcription [23,33,34].

In summary, the derived phytochemicals in GPE, analyzed using GC-MS, show promise as a natural alternative therapeutic strategy for UC due to its synergistic antioxidant, anti-inflammatory, and anti-apoptotic properties. These effects are attained via the modulation of NF-κB signaling and OS, suggesting a potential role in improving UC outcomes.

## 2. Results

### 2.1. Identification of Bioactive Compounds of GPE by GC-MS Analysis

**Figure 1:**
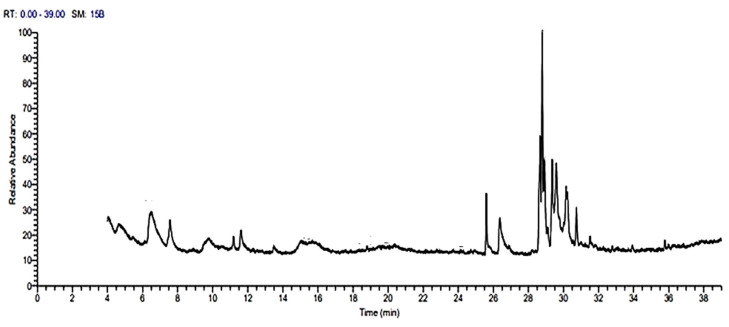
GC-MS total ion chromatogram (TIC) of analyzed GPE.

The GC–MS analysis of garlic peel extract (GPE), as presented in Table 1 and Figure 1, identified a diverse range of phytochemical constituents. The major detected components, based on peak area percentages, were fatty acid esters (62.47%), followed by fatty acids (10.36%), fatty acid derivatives (6.75%), vitamins (4.86%), steroids (3.39%), natural alcohols (2.72%), organosulfur compounds (2.64%), fatty aldehydes (2.51%), carotenoids (2.43%), sugars (0.99%), and glucosinolates (0.80%). In addition, several minor compounds were detected, including ketone derivatives, alkanes, oximes, pyranone derivatives, lactones, benzofuran derivatives, aromatic esters, and pyrrolopyridazine derivatives. A few other compounds were excluded due to unreliable identification by the database.

### 2.2. Effects of Inner Garlic Peel Ethanolic Extract (GPE) on Final Body Weight and Colon Length in AA-Induced UC Rats

**Figure 2:**
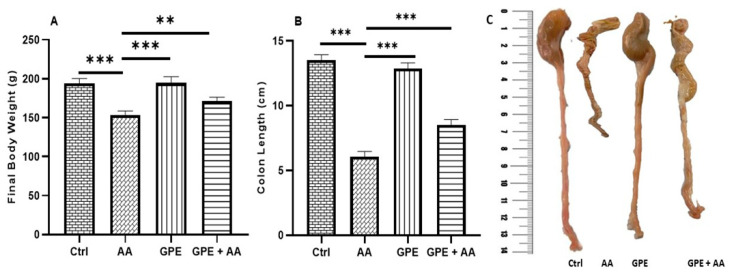
Effect of GPE on final body weight (**A**) and colon length (**B**) in AA-induced UC rats. Data expressed as mean values ± SD (n = 6/group). ** = *p* < 0.001 and *** = *p* < 0.0001 vs. the AA group. Representative pictures of colon length (**C**) in all experimental groups.

The final body weight (Figure 2A) and colon length (Figure 2B,C) in AA-induced UC rats (AA group) showed a highly significant decrease compared to the control (Ctrl) and the GPE-treated (GPE) groups. However, treatment of the AA-induced UC rats with GPE (GPE + AA group) resulted in a significant increase in final body weight and a highly significant increase in colon length. In spite of this, both the final body weight and colon length in the GPE + AA group were not restored completely back to normal, where a highly significant (*p* < 0.0001) decrease was observed in comparison with the Ctrl group.

### 2.3. Effects of Inner Garlic Peel Ethanolic Extract (GPE) on Pro- and Anti-Inflammatory Mediators and Transcription Factor NF-kB in AA-Induced UC Rats

**Figure 3:**
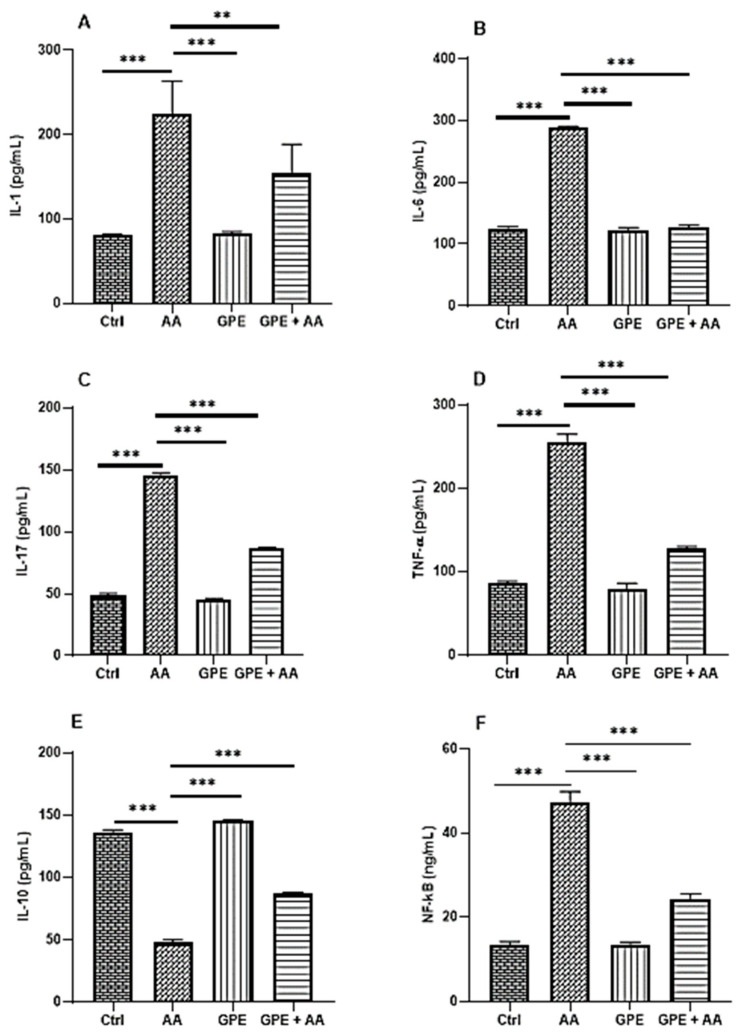
Effect of GPE on pro- and anti-inflammatory mediators (**A**) IL-1, (**B**) IL-6, (**C**) IL-17, (**D**) TNF-α, (**E**) IL-10, and (**F**) the transcription factor NF-κB in AA-induced UC rats. Data expressed as mean values ± SD (n = 6/group). ** = *p* < 0.001 and *** = *p* < 0.0001 vs. the AA group.

The levels of pro-inflammatory mediators IL-1 (Figure 3A), IL-6 (Figure 3B), IL-17 (Figure 3C), and TNF-α (Figure 3D) in the colon tissues of the AA group were highly significantly increased compared to the Ctrl group. However, treatment with GPE resulted in a significant (IL-1) to a highly significant (IL-6, IL-17, and TNF-α) decrease in the GPE + AA group. In addition to the pro-inflammatory mediators, a highly significant increase in the levels of the anti-inflammatory mediator IL-10 was observed in the GPE + AA group in comparison with the AA group (Figure 3E). Regarding NF-κB, known to be a pivotal mediator of inflammatory response, there was a highly significant increase in its level in the AA group compared to the Ctrl group. However, it was decreased in the GPE + AA group in comparison with the AA group with a statistically highly significant difference (Figure 3F). With the exception of IL-6, the observed change in the pro- and anti-inflammatory mediators as well as NF-kB levels upon treatment of the AA rats with GPE was not sufficient to completely restore the inflammatory mediators back to normal levels, where a significant difference (*p* < 0.001) was observed compared to the Crtl group.

### 2.4. Effects of Inner Garlic Peel Ethanolic Extract (GPE) on Oxidative Stress Biomarkers in AA-Induced UC Rats

**Figure 4:**
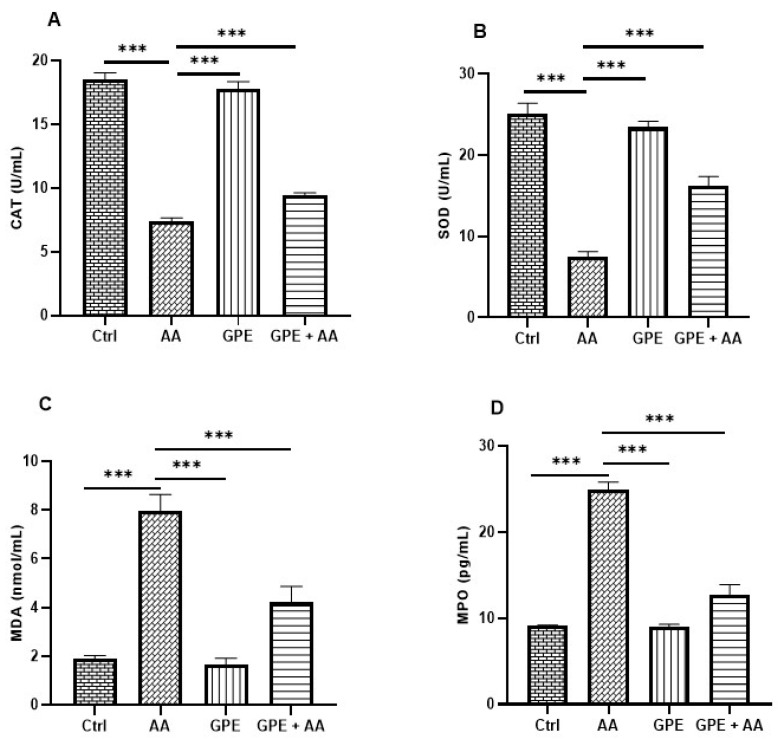
Effect of GPE on oxidative stress biomarkers (**A**) CAT, (**B**) SOD, (**C**) MDA, and (**D**) MPO in AA-induced UC rats. Data expressed as mean values ± SD (n = 6/group). *** = *p* < 0.0001 vs. the AA group.

In comparison with the Ctrl group, the activities of CAT (Figure 4A) and SOD (Figure 4B) in the AA group demonstrated a highly significant decrease. On the contrary, treatment with GPE resulted in a highly significant increase in CAT and SOD activities in the GPE + AA group compared to the AA group. In addition, a highly significant increase was observed in the MDA level in the AA group compared to the Ctrl group, whereas a highly significant decrease in the MDA level was observed in the GPE + AA group in comparison with the AA group (Figure 4C). Regarding the myeloperoxidase (MPO) level, a highly significant increase was observed in the AA group compared to the Ctrl group. However, the MPO level was reduced in the GPE + AA group in comparison with the AA group, with a statistically highly significant difference (Figure 4D). The detected change in CAT and SOD activities as well as MDA and MPO levels upon treatment of the AA rats with GPE was not sufficient to completely restore the biomarkers of OS back to normal levels, where a significant difference (*p* < 0.0001) was observed in comparison with the Ctrl group.

### 2.5. Effects of Inner Garlic Peel Ethanolic Extract (GPE) on Caspase-3 Levels in AA-Induced UC Rats

**Figure 5:**
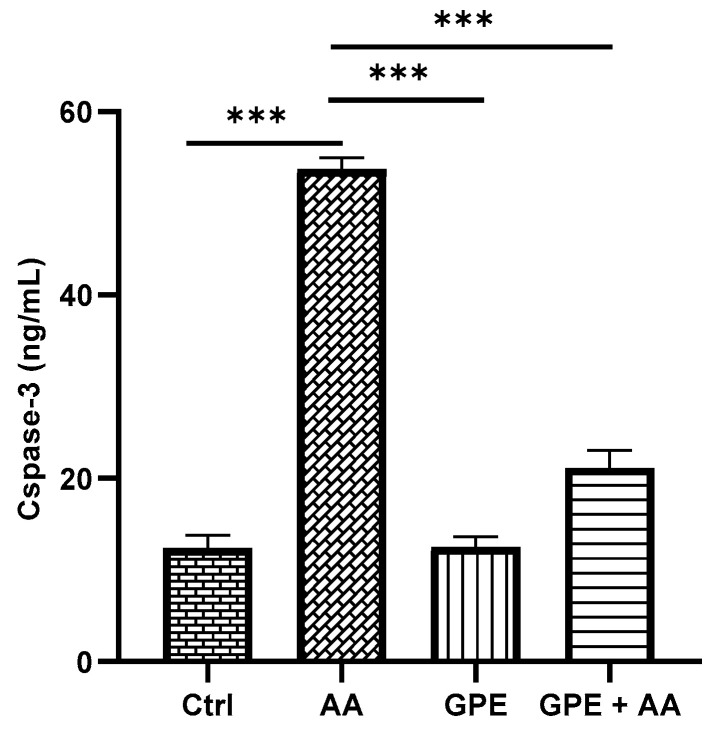
Effect of GPE on levels of caspase-3 in AA-induced UC rats. Data expressed as mean values ± SD (n = 6/group). *** = *p* < 0.0001 vs. the AA group.

In comparison with the Ctrl group, the level of caspase-3 in AA group showed a highly significant increase. On the contrary, treatment with GPE resulted in a highly significant decrease in the level of caspase-3 in the GPE + AA group compared to the AA group (Figure 5). However, the level of caspase-3 was not completely recovered back to normal levels in the GPE + AA group, where a significant difference (*p* < 0.001) was observed in comparison with the Ctrl group.

### 2.6. Effects of Inner Garlic Peel Ethanolic Extract (GPE) on Colon Histopathology in AA-Induced UC Rats

**Figure 6:**
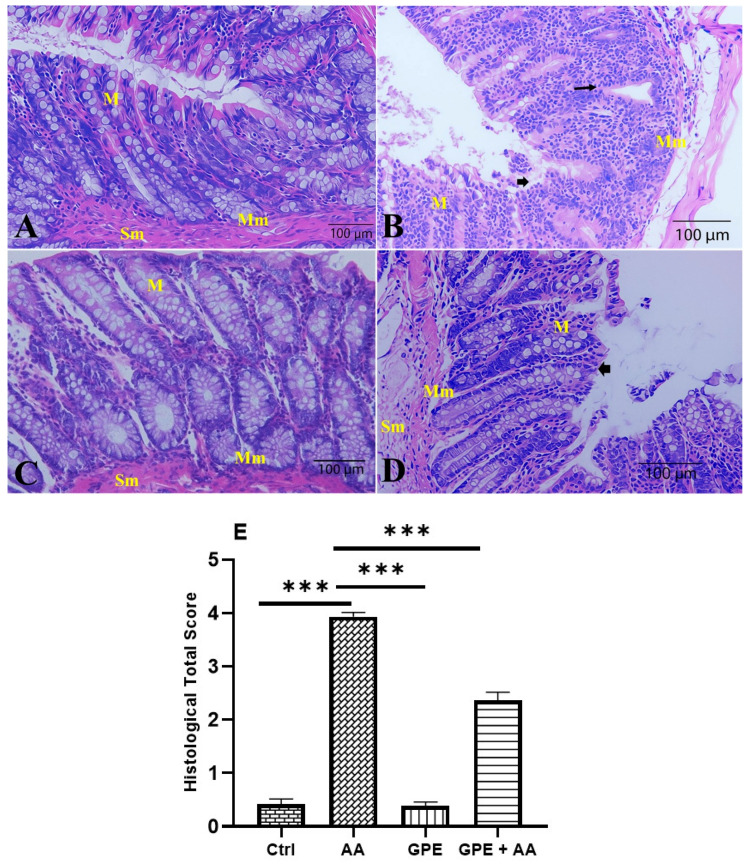
Photomicrograph of histologically analyzed H&E-stained colon sections in all experimental groups: (**A**) control rats (Ctrl group) showing normal mucosal structure with intact epithelial surface (M), muscular layer (Mm), and submucosa (Sm); (**B**) rats with AA-induced colitis (AA group) showing histopathological alterations evidenced by severe mucosal damage, loss of goblet cells (arrowheads), and necrosis of mucosa (M) associated with marked infiltration of mononuclear cells (arrow); (**C**) GPE-treated rats (GPE group) showing almost nearly normal mucosal structure (M), muscular layer (Mm), and submucosa (Sm); (**D**) GPE-treated rats with colitis (GPE + AA group) showing improvement in histological structure with slight loss of some surface columnar epithelial cells (arrowhead) and minimal necrosis of mucosa (M). Original magnification: 100×. (**E**) Scoring of histology sections. Data expressed as mean ± SD (n = 6/group). *** = *p* < 0.0001 vs. AA group.

As shown in Figure 6A, the H&E-stained sections of colonic tissues of the Ctrl group showed normal histological architecture of the mucosa with intact mucosal epithelium, an abundance of goblet cells, and no signs of inflammation. The colon tissues of the rats in the AA group, on the other hand, featured an altered histology evidenced by loss of surface columnar epithelial cells, necrosis of mucosa associated with severe infiltration of mononuclear inflammatory cells, and a reduced number of goblet cells (Figure 6B). On the contrary, sections of colon tissues of the GPE group showed nearly normal structures of the mucosa, muscular layer, and submucosa (Figure 6C). The histopathological examination of the sections of colon tissues of the GPE + AA group exhibited greater healing of ulcerous areas. Additionally, slight loss of some surface columnar epithelial cells as well as minimal necrosis of the mucosa, crypts, and goblet cells were recovered to the extent that they closely resemble those in the colon tissues of the Ctrl group (Figure 6D). When the GPE + AA group was compared to the AA group, the microscopic scores of the colon tissues’ histological sections revealed a highly significant decrease (Figure 6E).

## 3. Discussion

Ulcerative colitis (UC) is a chronic IBD that is characterized by relapsing episodes of mucosal inflammation and epithelial injury, often leading to severe long-term complications. Although conventional therapies, including 5-ASAs, CSs, IMMs, biologics, and Janus kinase (JAK) inhibitors have proven efficacy, their use is frequently limited by adverse effects, cytotoxicity, or diminished long-term effectiveness [63,64,65,66]. Consequently, there is growing interest in exploring safer, naturally derived therapeutic alternatives.

The present study provides compelling evidence for the therapeutic potential of GPE in ameliorating the pathophysiological features of UC in a rat model. GC-MS profiling revealed that GPE is rich in bioactive compounds, particularly fatty acid methyl esters (62.47%). The major esterified fatty acids identified, including 11-octadecenoic acid, linolenic acid, oleic acid, and palmitic acid methyl esters, have well-documented anti-inflammatory and antioxidant properties [67,68,69,70]. These esters can be hydrolyzed into free fatty acids (FFAs) such as polyunsaturated (PUFAs) and monounsaturated fatty acids (MUFAs), which are known to suppress the production of pro-inflammatory cytokines and promote mucosal healing [71,72,73]. Also, GC-MS analysis revealed that the GPE is rich in omega-3 fatty acids, particularly docosahexaenoic acid (DHA) and eicosapentaenoic acid (EPA), which are well known for their anti-inflammatory properties and may offer therapeutic benefits in inflammatory conditions such as IBD [48].

Moreover, vitamins identified in the extract, particularly ascorbic acid (vitamin C), have demonstrated the ability to inhibit the expression of NF-κB, COX-2, and iNOS, thereby alleviating OS and inflammation in DSS-induced colitis. The therapeutic potential of ascorbic acid in IBD is attributed not only to its strong antioxidant properties but also to its diverse biological activities [54]. As a powerful scavenger of ROS, vitamin C plays a key role in protecting the intestinal mucosa from damage [74,75].

A hallmark of UC is persistent inflammation driven by immune dysregulation and OS. In accordance with previous findings [26,33,76], our study showed that GPE treatment significantly downregulated the pro-inflammatory cytokines IL-1, IL-6, IL-17, and TNF-α, as well as NF-κB, a key player in inflammatory signaling. These cytokines are integral to both the initiation and perpetuation of the inflammatory response in IBDs, including UC [77,78]. The NF-κB signaling pathway plays a pivotal role in modulating immunological and inflammatory responses, rendering it one of the most prominent signaling pathways associated with UC [79,80,81]. Accordingly, activation of NF-kB and its downstream pro-inflammatory targets has been previously reported in AA-induced UC [82,83]. GPE treatment also led to a significant increase in IL-10, a key anti-inflammatory cytokine involved in limiting immune responses and maintaining epithelial homeostasis [84,85,86]. This suggests that GPE not only suppresses inflammation but may also promote immune resolution through the induction of regulatory cytokines.

Oxidative stress (OS) is another critical factor in UC pathogenesis [87,88]. Similar to our findings, several studies revealed signs of oxidative damage, demonstrated by decreased activities of the endogenous antioxidant enzymes CAT and SOD, as well as elevated markers of oxidative damage, including MPO activity and MDA levels, in AA-induced UC [6,83,89]. Moreover, it has been reported that the development of OS might be attributed to the excessive secretion of MPO and pro-inflammatory cytokines, such as TNF-α [90]. In the current study, GPE treatment significantly increased the activity of both CAT and SOD. On the other hand, MPO activity and MDA levels, representing LPO markers, as well as neutrophil infiltration, were significantly decreased. These findings underscore the antioxidant capacity of GPE in mitigating oxidative damage in colonic tissue. Indeed, the antioxidant activity of GPE has been previously reported [23,25,29].

In addition to the anti-inflammatory and antioxidant effects of GPE, it exhibited anti-apoptotic activity. Caspase-3, a key executioner enzyme in apoptosis, was significantly downregulated in GPE-treated groups. Apoptosis of colonic epithelial cells, frequently caused by OS, contributes to mucosal barrier disruption and the subsequent release of damage-associated molecular patterns (DAMPs), which trigger inflammation via pattern recognition receptors, including toll-like receptors (TLRs) [91,92,93]. The suppression of caspase-3 activity suggests that GPE helps preserve epithelial integrity and limits inflammation-induced tissue damage. This effect may be attributed to sulfur-containing compounds present in GPE, which possess known antioxidant and cytoprotective properties [33,34].

In the current study, histological analysis supported the biochemical findings. Colonic tissues from GPE-treated rats with colitis (GPE + AA group) showed significant healing of ulcerated mucosa, reduced inflammatory cell infiltration, and restored epithelial architecture, with only minimal residual necrosis. These results collectively demonstrate that GPE facilitates tissue regeneration by attenuating inflammation, OS, and epithelial apoptosis.

Compared to conventional UC treatments, GPE appears to offer several advantages. Commonly used for mild-to-moderate UC, 5-ASAs primarily reduce inflammation in the colonic epithelium [94] but lack significant antioxidant or anti-apoptotic properties. Corticosteroids (CSs) are effective anti-inflammatories but are generally restricted to short-term use due to severe systemic side effects that hinder their utilization for maintained remission [95,96]. GPE, with its favorable safety profile, may be more appropriate for long-term application. Immunomodulators (IMMs) suppress lymphocyte proliferation through cytotoxic mechanisms [97], while GPE appears to achieve immune modulation through natural, non-cytotoxic pathways. Moreover, it has been proposed that the use of IMMs should be limited to patients with milder UC who cannot maintain corticosteroid-free remission on 5-ASAs alone or in combination with TNF antagonists in patients with moderate-to-severe UC [98]. Biologics, such as anti-TNF-α agents, target specific inflammatory mediators [99,100], and GPE’s downregulation of TNF-α suggests it may function through a comparable, albeit broader, mechanism. Likewise, JAK inhibitors disrupt cytokine signaling at the intracellular level [101,102], whereas GPE appears to exert a more generalized anti-inflammatory effect.

Importantly, GPE’s ability to act directly on colonic tissue and reduce caspase-3 expression via localized delivery of bioactive compounds distinguishes it from conventional drugs, which often have systemic effects and broader immune suppression. This targeted action, along with its antioxidant, anti-inflammatory, and anti-apoptotic properties, highlights GPE’s potential as a safe and multifaceted therapeutic agent. A thorough comparison of the clinical characteristics and mechanisms of action of GPE with conventional UC treatments reveals significant distinctions that warrant attention (Table 2). Unlike conventional therapies, which often exhibit limited efficacy and potential toxicity, GPE presents a novel approach with the potential for more sustained therapeutic benefits.

## 4. Materials and Methods

### 4.1. Plant Material

Inner garlic peels were purchased from a private market at Al-Ahsa, Saudi Arabia. Peels were cleaned with water, dried in the shade, and then dried in an oven at no more than 50 °C until a constant weight was achieved, thus protecting sensitive active ingredients, as previously reported [103]. They were then ground to a fine powder (80-mesh sieves) and prepared to be used for ethanolic extraction.

### 4.2. Preparation of Garlic Peel Ethanolic Extract (GPE)

Garlic peel ethanolic extract (GPE) was prepared by soaking 400 g of garlic peel powder in ethyl alcohol (70%) overnight in the dark and then filtered through Whatman No. 1 filter paper. The filtrate was collected, dried by evaporation under reduced pressure, and then weighed [104]. The dried extract was stored in a dry sterilized airtight screw-cap tube at room temperature until further investigation. The dry mass of the concentrated extract served as the basis for all subsequent experiments.

### 4.3. Gas Chromatography–Mass Spectrometry (GC-MS) Analysis of GPE

The identification of the chemical composition of GPE was performed using a Trace GC1310-ISQ mass spectrometer (Thermo Scientific, Austin, TX, USA) endowed with a direct capillary column TG–5MS (30 m × 0.25 mm × 0.25 µm film thickness). Initially, the temperature of the column oven was set at 50 °C; this was followed by 5 °C/min increments until 230 °C, where it was held for 2 min. Finally, the temperature was raised to 290 °C at a rate of 30 °C/min and held for another 2 min. The temperature of both the injector and MS transfer line was retained at 250 °C and 260 °C, respectively. Helium (He), flowing at a constant rate of 1.0 mL/min, served as the carrier gas. The GC-MS setup involved the injection of 1.0 µL of diluted samples using an AS1300 autosampler in split injection mode with a 3 min solvent delay [105]. Collection of electron ionization (EI) mass spectra was carried out in full scan mode at a 70 eV ionization voltage, over a mass-to-charge ratio (*m*/*z*) range of 40–1000, and using an EI ion source set at 200 °C. The retention times and mass spectra of the identified components were compared with those of WILEY 09 and NIST 11 mass spectral libraries.

### 4.4. Animals

The 6–7-week-old male albino rats (150–160 g) utilized in the current study were obtained from an animal facility at King Faisal University, Saudi Arabia. The rats were maintained under environmentally controlled conditions, including a temperature of 24 ± 1 °C and a light/dark cycle of 12 h. In addition, a standard rodent diet and water were provided to the animals ad libitum. Acclimatization to such conditions was carried out over a period of 2 weeks before starting the experiment. All experimental procedures were performed in accordance with the protocol approved by the Research Ethics Committee at King Faisal University (Ref. No. KFU-REC-2025-FEB-ETHICS3047).

### 4.5. Ulcerative Colitis (UC) Induction

Ulcerative colitis (UC) was induced as previously reported [106]. In brief, animals were allowed access to water throughout the induction but were deprived of food 24 h before it started. Under light ether anesthesia, a medical-grade polyethylene tube was introduced 4.5 cm proximally to the anus verge and used to inject 2 mL of acid acetic solution (AA; 3% in normal saline—Sigma-Aldrich, St. Louis, MO, USA) into the colon’s lumen. The rats were maintained in a head-down position both during and for 30 s after instillation to prevent leakage of the solution. The rats in the control (Ctrl) group were exposed to the same treatment using 0.9% saline as a substitute for AA solution.

### 4.6. Experimental Design

Twenty-four rats were randomly divided into four groups (6 rats/group): Group I (control; Ctrl group): animals received normal saline rectally and orally for 4 weeks; Group II (Colitis; AA group): animals received a single rectal dose of acetic acid (AA) instillation; Group III (GPE group): animals received oral GPE (100 mg/kg), and at day 14, the rats were subjected to saline instillation and continued the treatment for 14 more days; and Group IV (GPE + AA group): animals received oral GPE, and at day 14, the rats were subjected to UC induction as in Group II and continued the treatment for 14 more days. Upon completion of the experiment, rats from all experimental groups were euthanized using an overdose of ether. For enzyme-linked immunosorbent analysis (ELISA), the proximal colonic segments were promptly kept at −80 °C, while the distal colonic portions were prepared for histopathological investigation.

### 4.7. Preparation of Colonic Tissue Homogenates

The excised colon tissues were thoroughly rinsed with ice-cold phosphate-buffered saline (PBS; 0.01 mol/L, pH 7.0–7.2—Thermo Fisher Scientific, Waltham, MA, USA) to remove any residual blood and then weighed prior to homogenization. The weighed tissues were minced into small pieces; this was followed by their homogenization on ice in 5–10 mL PBS using a glass homogenizer. The obtained suspensions were sonicated using an ultrasonic cell disrupter that further broke the cell membranes. Next, the homogenates were centrifuged at 5000× *g* for 15 min at 4 °C using a Thermo Scientific™ Heraeus™ Multifuge X3R centrifuge (Thermo Fisher Scientific, Waltham, MA, USA), and the resulting supernatants were further used in ELISA assays.

### 4.8. Immunological and Molecular Assays

ELISA kits (My Bio-Source, Inc., San Diego, CA, USA) were used to assess the levels of IL-1 (Cat. No.: MBS264984), IL-6 (Cat. No.: MBS495243), IL-10 (Cat. No.: MBS2503826), IL-17 (Cat. No.: MBS723014), TNF-α (Cat. No.: MBS267737), NF-κB (Cat. No.: MBS453975), and Caspase 3 (Cat. No.: MBS4539) according to the manufacturer’s manual.

### 4.9. Oxidative Stress Assays

ELISA kits (My Bio-Source, Inc., San Diego, CA, USA) were used to assess the OS biomarkers, including the activity of the endogenous antioxidant enzymes CAT (Cat. No.: MBS006963) and SOD (Cat. No.: MBS036924) as well as the levels of MPO (Cat. No.: MBS450065) as described by the manufacturer’s manual. Levels of malondialdehyde (MDA), a marker of LPO, were determined by the thiobarbituric acid (TBA) test at 532 nm as previously described [107].

### 4.10. Histopathological Examination

The distal portions obtained from the excised colons were processed for histopathological examination as follows: First, the tissues were fixed in a 10% formaldehyde solution (Merck, Darmstadt, Germany) for 24 h at room temperature. They were then embedded in paraffin wax and sectioned to obtain 6 µm thick paraffin sections. Staining was carried out with hematoxylin and eosin (H&E—Abcam, Cambridge, UK), and sections were subsequently examined under a light microscope (Nikon Corporation, Tokyo, Japan).

The extent of colon ulceration was assessed on the basis of the observed inflammatory response. Accordingly, the inflammatory response was quantified using a scoring system that ranged from 0 to 4, as indicated: 0, no observed inflammation; 1, a low degree of inflammation with scattered infiltrating mononuclear cells; 2, a moderate degree of inflammation with multiple infiltrating mononuclear cells and a slight loss of some surface columnar epithelial cells; 3, a high degree of inflammation together with a high degree of mononuclear cell infiltration and loss of crypt structure; 4, optimal severity of inflammation with loss of some surface columnar epithelial cells and mucosal necrosis [4].

### 4.11. Statistical Analysis

Data were represented as mean values ± standard deviation (SD). One-way analysis of variance (ANOVA), followed by Tukey’s multiple comparisons post hoc test, was used to analyze the statistical difference (*p* < 0.05) among groups using GraphPad Prism 8 Software, version 8.0.2 (GraphPad Software, San Diego, CA, USA).

## 5. Conclusions

To our knowledge, the findings from this study suggest for the first time that GPE could serve as a valuable adjunct or alternative to conventional therapies for UC. Its broad-spectrum biological activity, coupled with a favorable safety profile, warrants further investigation in clinical trials to confirm its efficacy in human patients.

## Figures and Tables

**Table 1:** Chemical compounds identified in GPE by GC-MS analysis.

Name of Detected Compound	RT (min)	MF	MW	A (%)	CAS #	Biological Role(s)
Fatty acid esters						
Heptanoic acid, 6-oxo-,methyl ester	6.51	C_8_H_14_O_3_	158	1.91	2046-21-1	Not reported
Palmitic acid, methyl ester	25.60	C_17_H_34_O_2_	270	5.26	112-39 -0	Anti-inflammatory [35]
α-Linolenic acid, methyl ester, omega-3	28.66	C_19_H_32_O_2_	292	9.58	7361-80-0	Antioxidant; anti-inflammatory [36,37]
11-Octadecenoic acid, methyl ester	28.79	C_19_H_36_O_2_	296	15.87	52380-33-3	Antimicrobial; anti-inflammatory; antioxidant [38]
10-Octadecenoic acid, methyl ester	28.91	C_19_H_36_O_2_	296	6.97	13481-95-3	Antimicrobial; antioxidant [39]
6,9-Octadecadiynoic acid, methyl ester	29.10	C_19_H_30_O_2_	290	1.02	56847-03-1	Anticancer; antimicrobial [40]
Stearic acid, methyl ester	29.35	C_19_H_38_O_2_	298	7.55	112-61-8	Antioxidant; antifungal [41]
Nonanoic acid, methyl ester	29.58	C_10_H_20_O_2_	172	6.18	1731-84-6	Not reported
4,7-Octadecadiynoic acid, methyl ester	30.15	C_19_H_30_O_2_	290	3.69	18202-20-5	Contribution to antioxidant and cytotoxic activities [42]
7,10-Octadecadienoic acid, methyl ester	30.23	C_19_H_34_O_2_	294	3.62	56554-24-6	Might enhance immune function [43]
Stearic acid, 3-(octadecyloxy) propyl ester	35.70	C_39_H_78_O_3_	594	0.45	17367-40-7	Not reported
Cyclopropanedodecanoic acid, 2-octyl-, methyl ester	35.98	C_24_H_46_O_2_	366	0.37	10152-65-5	Antioxidant; anti-inflammatory; reduction in lipid peroxidation [44]
Fatty acids						
Oleic acid (omega-9 fatty acid)	4.04	C_18_H_34_O_2_	282	0.62	112-80-1	Antioxidant [45]
Eicosapentaenoic acid (fish omega-3)	29.58	C_20_H_30_O_2_	302	6.18	10417-94-4	Anti-inflammatory; antioxidant [5,46,47,48]
Docosahexaenoic acid (omega 3)	30.47	C_22_H_32_O_2_	328	3.56	NA	Anti-inflammatory; antioxidant [46,47,49]
Fatty acid derivatives						
6-Dodecanone (derived from lauric acid)	6.41	C_12_H_18_O	178	3.23	NA	Not reported
2-Monolinolenin (derived from linolenic acid)	24.65	C_27_H_52_O_4_Si_2_	496	2.91	55521-23-8	Antibacterial; antifungal [50]
2-Bromotetradecanoic acid (derived from myristic acid)	32.78	C_14_H_27_BrO_2_	306	0.61	10520-81-7	Antifungal [51]
Vitamins						
Thiamine hydrochloride (vitamin B_1_)	23.83	C_12_H_17_ClN_4_OS	338	0.32	NA	Antioxidant [52]
L-Ascorbic acid 2,6-dihexadecanoate	26.36	C_38_H_68_O_8_	652	4.18	28474-90-0	Antioxidant [53,54]
Tocopherol (Vitamin E)	29.50	C_29_H_50_O_2_	431	0.36	NA	Mitigation of colitis [55]
Steroids						
β-sitosterol	11.20	C_29_H_50_O	415	3.18	NA	Ameliorates colitis [56]
Ethyl iso-allocholate	31.78	C_26_H_44_O_5_	436	0.21	47676-48-2	Anti-apoptotic [57]
Natural alcohols						
Hexahydrofarnesol (sesquiterpene alcohol)	9.92	C_15_H_32_O	228	0.33	6750-34-1	Not reported
1-Heptatriacotanol (fatty alcohol)	30.01	C_37_H_76_O	536	2.39	105794-58-9	Antimicrobial [58]
Organosulfur compounds						
Ethanimidothioic acid (derived from sulfur amino acid)	4.64	C_7_H_13_N_3_O_3_S	219	1.21	23135-22-0	Not reported
Cysteamine sulphonic acid	11.18	C_2_H_7_NO_3_S_2_	157	1.43	2937-53-3	Supporting mucosal healing in colitis [59]
Fatty aldehydes						
11-Octadecenal	4.12	C_18_H_34_O	266	0.28	56554-95-1	Not reported
Butanedial	4.81	C_4_H_6_O_2_	86	0.18	638-37 -9	Not reported
3,5-Heptadienal, 2-ethylidene-6-methyl	11.6	C_10_H_14_O	150	2.05	99172-18-6	Antioxidant; antifungal [60]
Carotenoids						
ψ,ψ-Carotene	30.99	C_42_H_64_O_2_	600	2.43	13833-01-7	Antioxidant [61]
Sugars						
Melezitose	9.81	C_18_H_32_O_16_	504	0.65	597-12-6	Not reported
Arabinitol	36.51	C_15_H_22_O_10_	362	0.34	26674-23-7	
Glucosinolates						
Glucobrassicin	20.38	C_16_H_20_N_2_O_9_S_2_	448	0.8	4356-52-9	Modulation of inflammatory mediators in IBD [62]

RT = retention time; MF = molecular formula; MW = molecular weight; A = area; CAS # = chemical abstract service number.

**Table 2:** Comparative analysis of GPE and conventional UC therapies.

Therapeutic Agent	Key Mechanisms and Benefits	Main Limitations	Comparative Advantages of GPE	Reference(s)
GPE	Multimodal: antioxidant, anti-inflammatory (↓TNF-α), anti-apoptotic (↓caspase-3); localized delivery	Requires further clinical validation	Targeted action; minimal systemic effects; suitable for chronic use	Our study
Aminosalicylates (5-ASA)	Topical anti-inflammatory (epithelial COX/LOX inhibition)	No antioxidant/anti-apoptotic activity	Broader cytoprotection beyond inflammation control	[94]
Corticosteroids (CSs)	Systemic immunosuppression (↓NF-κB/cytokines)	Severe metabolic effects; contraindicated for maintenance	Safer long-term profile without steroidogenic effects	[95,96]
Immunomodulators (IMMs)	Cytotoxic lymphocyte suppression	Infection/malignancy risks	Non-cytotoxic immune regulation via natural pathways	[97]
Biologics (Anti-TNF)	Neutralize TNF-α, effective for moderate-to-severe UC	Immunogenicity; high cost	Natural TNF-α downregulation with lower immunogenicity	[99,100]
JAK Inhibitors	Block intracellular cytokine signaling (JAK/STAT pathway)	Thromboembolic risk; pan-cytokine inhibition	Selective anti-inflammatory action without broad immunosuppression	[101,102]

## Data Availability

The data supporting the results of the present study are included within this article and its Supplementary Materials.

## Supplementary Materials

The following supporting information can be downloaded at: https://www.mdpi.com/article/10.3390/ph18070969/s1, Supplementary File S1: Zip-Document (Raw Data).

## Abbreviations

The following abbreviations are used in this manuscript:

AA	Acetic acid
5-ASAs	5-aminosalicylates
CAT	Catalase
COX-2	Cyclooxygenase-2
CSs	Corticosteroids
DAMPs	Damage-associated molecular patterns
DAS	Diallyl sulfide
DHA	Docosahexaenoic acid
DSS	Dextran sulfate sodium
EPA	Eicosapentaenoic acid
FFAs	Free fatty acids
GPE	Garlic peel extract
GC-MS	Gas chromatography–mass spectrometry
IMMs	Immunomodulators
IBD	Inflammatory bowel disease
IL-1	Interleukin 1
IL-6	Interleukin 6
IL-10	Interleukin 10
IL-17	Interleukin 17
iNOS	Inducible nitric oxide synthase
JAK	Janus kinase
LPO	Lipid peroxidation
MDA	Malondialdehyde
MPO	Myeloperoxidase
MUFAs	Monounsaturated fatty acids
NF-κB	Nuclear factor kappa B
OSCs	Organosulfur-containing compounds
OS	Oxidative stress
PUFAs	Polyunsaturated fatty acids
ROS	Reactive oxygen species
SAC	S-allyl-L-cysteine
SOD	Superoxide dismutase
TLRs	Toll-like receptors
TNF-α	Tumor necrosis factor-alpha
UC	Ulcerative colitis
