# Supplementary material for: Garlic Peel-Derived Phytochemicals Using GC-MS: Antioxidant, Anti-Inflammatory, and Anti-Apoptotic Effects in Ulcerative Colitis Rat Model
Source: Pharmaceuticals (Basel). 2025 Jun 27;18(7):969. doi: 10.3390/ph18070969 (PMC12298587; doi:10.3390/ph18070969)
Supplement: Supplementary file 1 [file pharmaceuticals-18-00969-s001.zip › pharmaceuticals-3710317-supplementary/GC-MS_Raw Data.pdf]

My GC-MS Report

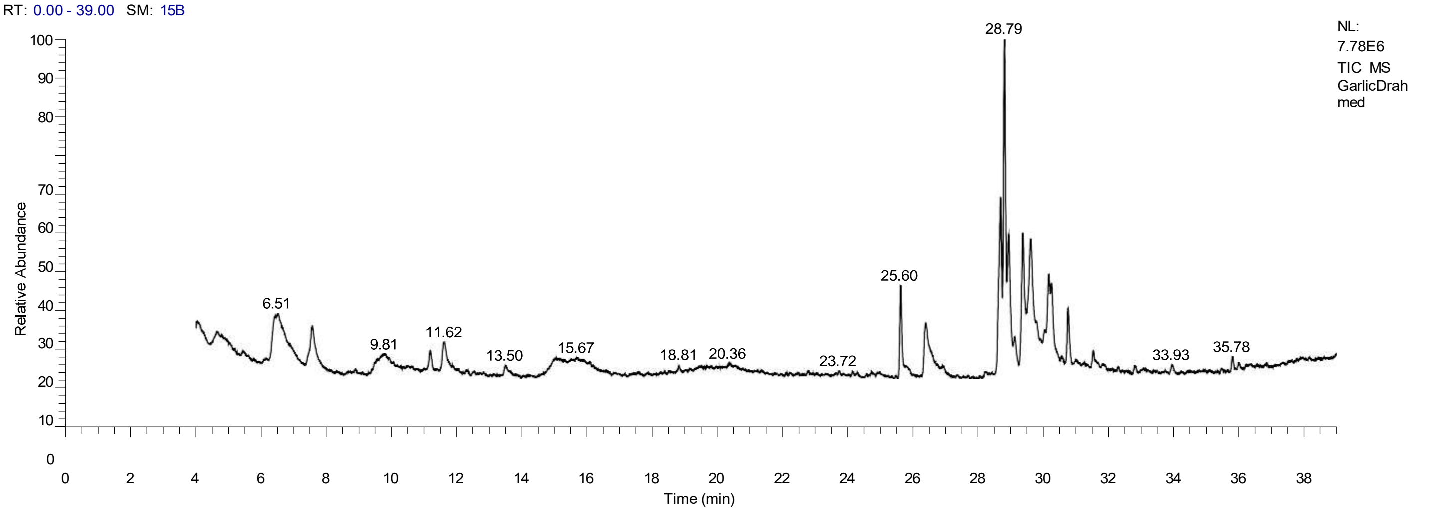

RT	Area %	Peak Area	Peak Height
4.04	0.62	874043.42	168029.05
4.12	0.28	402270.03	144616.00
4.64	1.21	1714468.28	198425.18
4.81	0.18	261671.78	105003.15
4.97	0.13	176932.60	98508.24
5.45	0.42	599495.08	101653.05
6.41	3.23	4572052.48	564864.94
6.51	1.91	2705510.46	485431.09
7.56	3.88	5492608.92	649266.61
8.88	0.24	340923.13	94759.87
9.73	0.40	566014.01	135371.20
9.81	0.69	973703.73	170225.33
9.92	0.33	469736.97	98709.69
11.18	1.43	2023752.22	409699.75
11.20	3.18	5011001.15	1201223.18
11.60	2.05	2898872.93	452805.85
12.28	0.25	348215.03	103261.86
13.50	0.65	916079.68	165409.99
14.97	0.61	858098.18	150051.52
15.02	0.44	618752.77	160244.83
15.11	0.27	384051.77	96383.48
18.81	0.49	696883.66	148120.04
20.38	0.33	466242.57	92377.99
22.47	0.13	177413.52	100808.18
22.77	0.30	421812.83	107144.30
23.73	0.32	447714.92	105535.91
23.83	0.32	447714.92	105535.91
24.14	0.38	532273.84	132228.94
24.30	0.35	492695.63	92714.16
24.56	0.17	245909.16	91746.32
24.73	0.41	576657.84	98030.02
24.88	0.20	282162.62	93097.35
25.45	0.23	328904.30	100668.49
25.60	5.26	7440498.86	1984171.35
26.36	4.18	5908836.66	846690.14
26.91	0.41	587037.88	117609.28
28.18	0.45	634046.55	108824.58
28.66	9.58	13564202.07	2831273.71
28.78	15.87	22458609.74	6224454.02
28.91	6.97	9862493.46	1999249.69
29.10	1.02	1444412.88	354860.98

RT	Area %	Peak Area	Peak Height
29.35	7.55	10683598.88	2317846.41
29.50	0.36	501102.31	117340.42
29.58	6.18	8745673.34	1495905.16
29.78	0.36	509212.90	157098.07

RT	Area %	Peak Area	Peak Height
30.01	0.39	548460.53	173431.86
30.15	3.69	5225463.51	1267012.48
30.23	3.62	5124464.43	1148687.81
30.53	0.38	531532.38	129804.46
30.74	3.56	5041271.15	1226923.88
30.99	0.43	609663.33	115652.60
31.22	0.24	342186.52	98699.55
31.51	1.45	2056206.61	392787.92
31.78	0.21	296145.79	110681.37
31.84	0.35	501069.61	110001.47
32.28	0.30	427265.10	111450.83
32.78	0.61	857667.54	161603.19
33.06	0.29	404797.96	100303.21
33.12	0.18	259773.72	92614.64
33.93	0.93	1319154.38	211983.64
34.91	0.17	244498.90	101382.00
35.42	0.14	203912.17	107871.93
35.46	0.20	280698.00	103944.13
35.78	0.94	1332653.59	344409.25
35.98	0.37	516829.04	134225.44
36.30	0.33	473062.89	107510.59
36.34	0.25	348926.36	106577.77
36.51	0.34	484222.72	112816.14
38.96	0.27	388471.10.	141484.78

NO	Compound	RT (min)	MF	MW	A (%)	CAS #
1.	Oleic acid (omega-9)	4.04	C18H34O2	282	0.62	112-80-1
2.	11-Octadecenal	4.12	C18H34O	266	0.28	56554-95-1
3.	Ethanimidothioic acid	4.64	C7H13N3O3S	219	1.21	23135-22-0
4.	Butanedial	4.81	C4H6O2	86	0.18	638-37-9
5.	6-Dodecanone	6.41	C12H18O	178	3.23	NA
6.	Heptanoic acid, 6-oxo-, methyl ester	6.51	C8H14O3	158	1.91	2046-21-1
7.	Melezitose	9.81	C18H32O16	504	0.69	597-12-6
8.	Hexahydrofarnesol	9.92	C15H32O	228	0.33	6750-34-1
9.	Cysteamine sulphonic acid	11.18	C2H7NO3S2	157	1.43	2937-53-3
10.	β-Sitosterol	11.20	C29H50O	415	3.18	NA
11.	3,5-Heptadienal, 2-ethylidene-6-methyl	11.60	C10H14O	150	2.05	99172-18-6
12.	Glucobrassicin	20.38	C16H20N2O9S2	448	0.80	4356-52-9
13.	Thiamin hydrochloride (Vitamin B1)	23.83	C12H17ClN4OS	338	0.32	NA
14.	2-Monolinolenin	24.65	C27H52O4Si2	496	2.91	55521-23-8
15.	Palmitic acid, methyl ester	25.60	C17H34O2	270	5.26	112-39-0
16.	L-Ascorbic acid 2,6-dihexadecanoate	26.36	C38H68O8	652	4.18	28474-90-0
17.	α-Linolenic acid, methyl ester (omega-3)	28.66	C19H32O2	292	9.58	7361-80-0
18.	11-Octadecenoic acid, methyl ester	28.79	C19H36O2	296	15.87	52380-33-3
19.	10-Octadecenoic acid, methyl ester	28.91	C19H36O2	296	6.97	13481-95-3
20.	High-Oleic safflower oil	29.10	C21H22O11	450	1.02	8001-23-8
21.	Stearic acid, methyl ester	29.35	C19H38O2	298	7.55	112-61-8
22.	Tocopherol (Vitamin E)	29.50	C29H50O2	431	0.36	NA
23.	Nonanoic acid, methyl ester	29.58	C10H20O2	172	6.18	1731-84-6
24.	Ecosapentaenoic acid (omega-3)	29.58	C20H30O2	302	6.18	10417-94-4
25.	4,7-Octadecadiynoic acid, methyl ester	30.15	C19H30O2	290	3.69	18202-20-5
26.	7,10-Octadecadienoic acid, methyl ester	30.23	C19H34O2	294	3.62	56554-24-6

27.	1-Heptatriacotanol	30.01	C37H76O	536	2.39	105794-58-9
28.	Docosahexaenoic acid (omega-3)	30.47	C22H32O2	328	3.56	NA
29.	Cis-psi,psi-carotene	30.99	C42H64O2	600	2.43	13833-01-7
30.	Ethyl iso-allocholate	31.78	C26H44O5	436	0.21	47676-48-2
31.	2-Bromotetradecanoic acid	32.78	C14H27BrO2	306	0.61	10520-81-7
32.	Stearic acid, 3-(octadecyloxy) propyl ester	35.70	C39H78O3	594	0.45	17367-40-7
33.	Cyclopropanedodecanoic acid, 2-octyl-, methyl ester	35.98	C24H46O2	366	0.37	10152-65-5
34.	Arabinitol	36.51	C15H22O10	362	0.34	26674-23-7
