# Supplementary material for: Garlic Peel-Derived Phytochemicals Using GC-MS: Antioxidant, Anti-Inflammatory, and Anti-Apoptotic Effects in Ulcerative Colitis Rat Model
Source: Pharmaceuticals (Basel). 2025 Jun 27;18(7):969. doi: 10.3390/ph18070969 (PMC12298587; doi:10.3390/ph18070969)

Histopathological results of Colon

Control

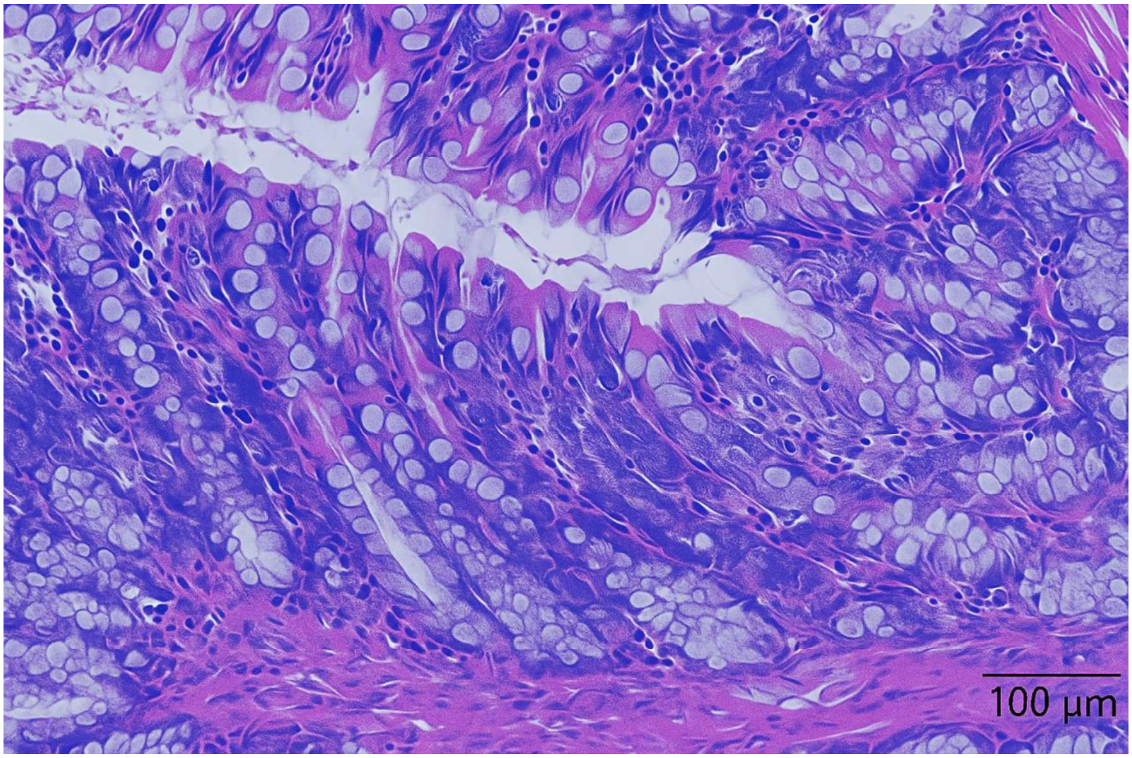

AA-induced Colitis

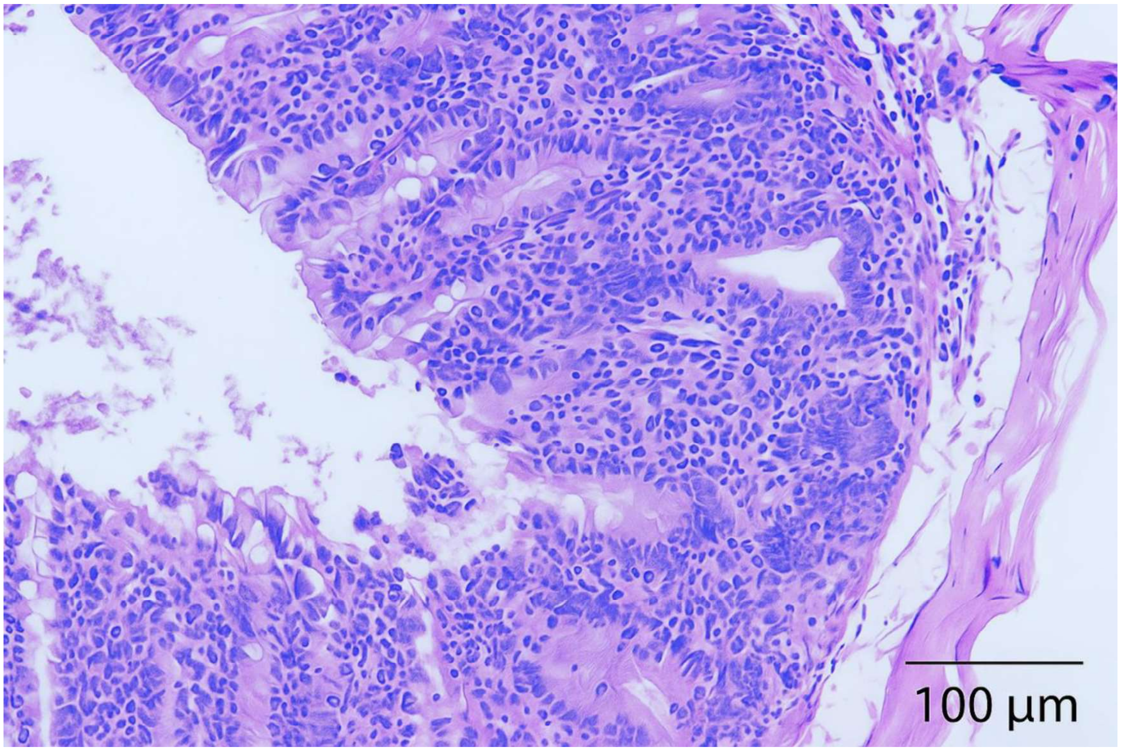

Garlic Peel Extract Only

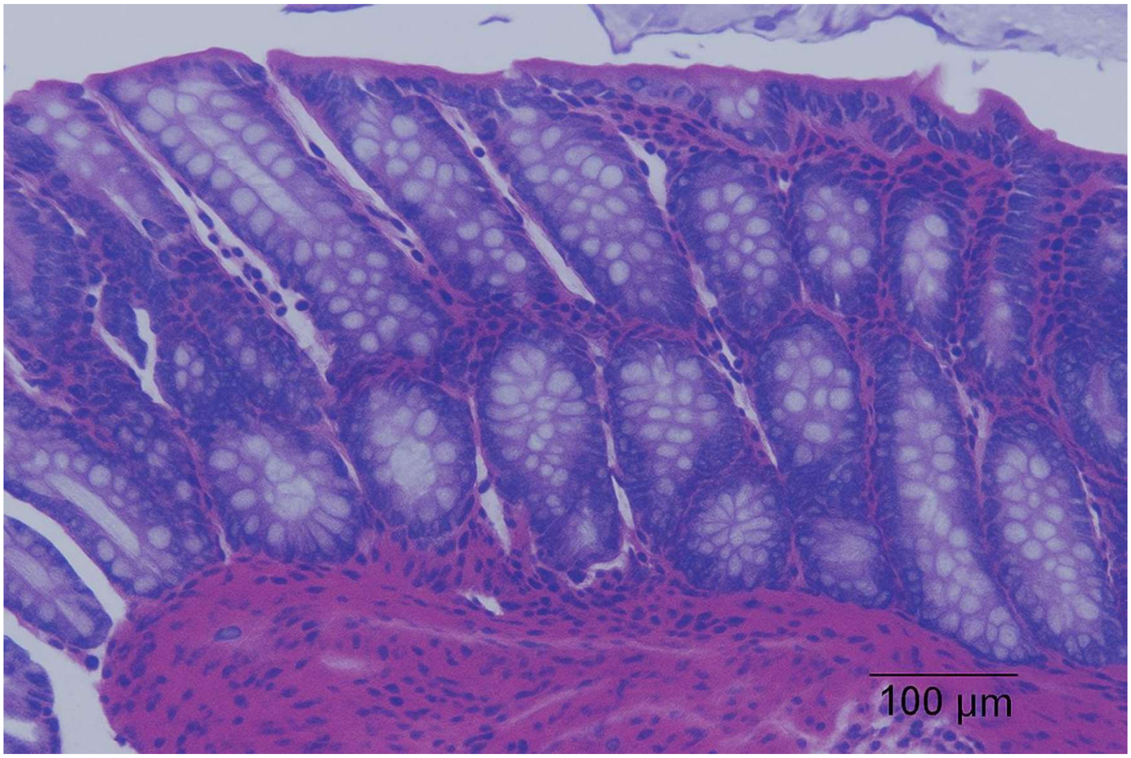

AA+GPE

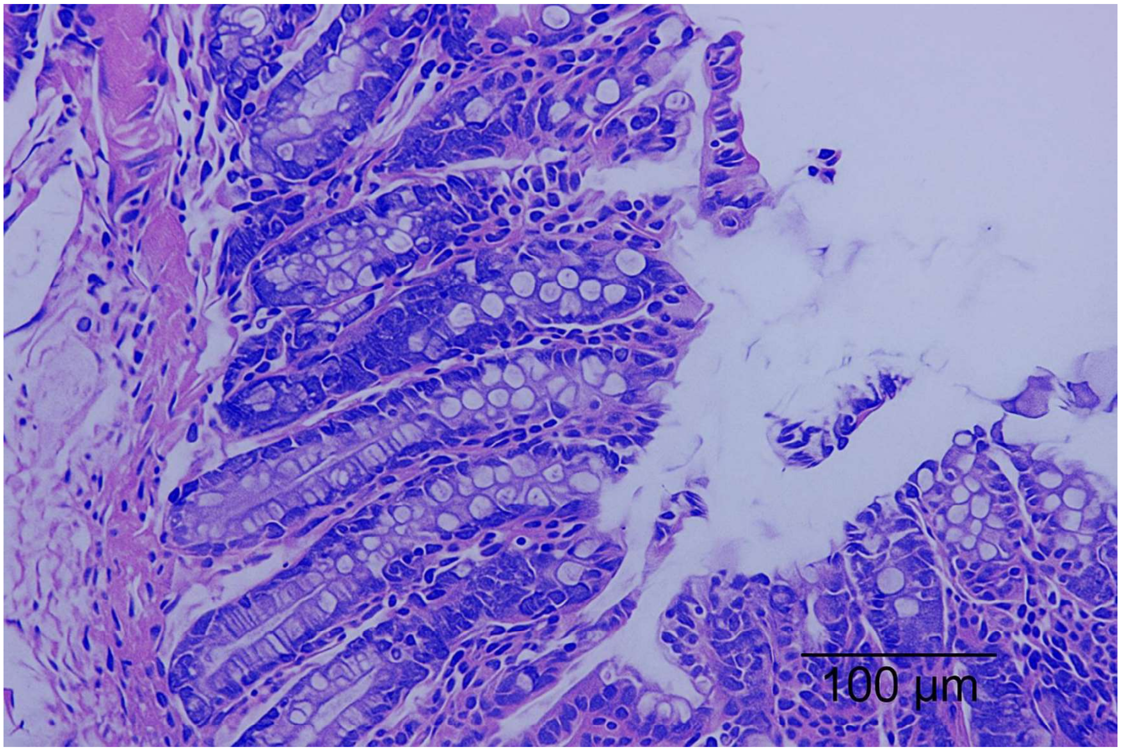

Supplement: Supplementary file 1 [file pharmaceuticals-18-00969-s001.zip › pharmaceuticals-3710317-supplementary/Original Histology Photos.pdf]
